# Navigating ‘k-land’: a qualitative exploration of participants’ experiences of ketamine-assisted psychotherapy for methamphetamine use disorder

**DOI:** 10.3389/fpsyt.2026.1873497

**Published:** 2026-06-24

**Authors:** Kathryn Fletcher, Nadine Ezard, Krista J. Siefried, Sophie van der Helder, Jack Freestone, Jonathan Brett, Robert May, Liam Acheson, Brendan Clifford

**Affiliations:** 1 The National Centre for Clinical Research on Emerging Drugs, University of New South Wales, Sydney, NSW, Australia; 2 Alcohol and Drug Service, St Vincent’s Hospital, Sydney, NSW, Australia; 3 The National Drug and Alcohol Research Centre, University of New South Wales, Sydney, NSW, Australia; 4 Drug and Alcohol Clinical Research and Improvement Network, NSW Health, Sydney, NSW, Australia; 5 St Vincent’s Clinical School, School of Population Health, University of New South Wales, Sydney, NSW, Australia; 6 Clinical Pharmacology and Toxicology, St Vincent’s Hospital Sydney, Sydney, NSW, Australia

**Keywords:** addiction treatment, ketamine-assisted psychotherapy (KAP), methamphetamine use disorder (MAUD), qualitative research, substance use disorder, ketamine, psychotherapy

## Abstract

**Background:**

Methamphetamine use disorder (MAUD) is associated with substantial psychiatric and physical morbidity, and current treatment options remain limited. Ketamine-assisted psychotherapy (KAP) has shown promise in substance use disorders, but little is known about how individuals experience and interpret this intervention, particularly in MAUD. Qualitative investigation may provide insight into perceived mechanisms, acceptability, and factors influencing engagement. This study aimed to explore how participants experienced, interpreted, and evaluated KAP for MAUD.

**Methods:**

This qualitative study was embedded within an open-label pilot trial of KAP for MAUD. Fourteen participants who completed core components of the intervention undertook semi-structured interviews following treatment. Interviews explored motivations for participation, experiences of ketamine dosing and psychotherapy, and perceptions of change. Data were analysed using reflexive thematic analysis.

**Results:**

Four interrelated themes were generated: (1) treatment entry, characterised by prior treatment experience with limited sustained benefit and pragmatic openness to a novel approach; (2) a structured and supportive context for engagement, highlighting the importance of relational safety and clinical containment; (3) altered states and psychological shifts, in which participants described reduced emotional and cognitive reactivity following ketamine sessions; and (4) translation of psychological shifts into behavioural change, describing variable and contingent changes in methamphetamine use. Participants described ketamine as creating a temporary state characterised by reduced emotional and cognitive reactivity and enhanced receptivity, which we interpret as ‘psychological space’. This state appeared to support engagement with psychotherapy, although behavioural change was variable and contingent on ongoing therapeutic engagement, personal motivation, and contextual factors. Acceptability was generally high within a supportive clinical environment. Participants expressed uncertainty regarding whether changes were attributable to ketamine, psychotherapy, or contextual factors.

**Conclusions:**

KAP for MAUD was experienced as a multi-stage, context-dependent process rather than a stand-alone pharmacological treatment. Ketamine was characterised as facilitating a temporary state of ‘psychological space’ that appeared to support engagement with psychotherapy, while sustained change appeared to depend on the integration of these experiences into ongoing cognitive and behavioural processes. These findings support a model in which pharmacological, psychotherapeutic and contextual factors interact, and highlight the need for further research to clarify mechanisms and optimise intervention design.

## 1 Introduction

Methamphetamine is an amphetamine-type stimulant associated with euphoria, increased energy and arousal, heightened behavioural activation and engagement with the environment, reduced inhibitions, and increased sexual drive and pleasure (1, 2). While these acute effects may contribute to its reinforcing properties, patterns of methamphetamine use are shaped by individual, relational, and environmental factors (2).

Substance use disorders are commonly conceptualised as involving compulsive substance use despite harm, partly driven by dysregulation in cortico-striatal-limbic circuits involved in reward, salience and executive control (3, 4). Contemporary models further conceptualise substance use as a disorder of pathological learning and maladaptive neuroplasticity, whereby repeated substance use produces enduring circuit-level changes that reinforce drug-related habits and cue responsiveness (5). While psychotherapies can facilitate corrective learning processes, interventions that temporarily enhance neuroplasticity and increase sensitivity to environmental input may offer additional therapeutic benefit, particularly when delivered in conjunction with structured psychological treatment.

Ketamine, an N-methyl-D-aspartate (NMDA) receptor antagonist, modulates glutamatergic signalling and produces rapid antidepressant effects (6, 7). Ketamine has demonstrated rapid effects across a range of affective symptoms, including depression and suicidality, highlighting broader effects on emotional processing and affective dysregulation alongside its antidepressant properties (8, 9). Preclinical and clinical studies suggest that subanaesthetic doses of ketamine enhance synaptic plasticity and temporarily increase sensitivity to environmental input and learning processes (7). In substance use disorders, it has been hypothesised that these effects may facilitate extinction learning (i.e., the weakening of conditioned associations between drug-related cues and drug use), weaken maladaptive reward associations, and enhance receptivity to behavioural interventions delivered in close temporal proximity to ketamine dosing (10).

In ketamine-assisted psychotherapy (KAP), ketamine is proposed to prolong and enhance psychotherapeutic effects by promoting neuroplasticity and increasing responsiveness to therapeutic input (11, 12). These processes may facilitate emotional learning, evoke subjectively salient experiences, reduce defensiveness and enhance treatment engagement (11). A recent systematic review found that ketamine, particularly when combined with psychotherapy, was associated with increased abstinence rates, reduced craving, delayed relapse, and greater motivation to change across alcohol, cocaine, opioid, and cannabis use disorders (13). However, the literature remains preliminary, characterised by small sample sizes, methodological heterogeneity, and limited clarity regarding mechanisms of action. Reviews of KAP similarly highlight the scarcity of randomised trials, variability in therapeutic models, and the need for more rigorous investigation of how ketamine and psychotherapy interact (14).

Beyond its neurobiological effects, ketamine produces dose-dependent alterations in consciousness. Subanaesthetic doses are associated with dissociative and perceptual changes (9), with low-dose euphoric and dissociative effects sometimes referred to colloquially as ‘k-land’, in contrast to the more profound dissociative states described as a ‘k-hole’ at higher doses (15). These experiences can range from feelings of detachment and perceptual distortion to states of emotional intensity and may at times be challenging or anxiety-provoking (16, 17). How individuals experience and interpret these effects may shape therapeutic processes, including emotional processing, cognitive flexibility, and shifts in self-concept and personal meaning (18). Therapeutic support and a strong therapeutic alliance may be particularly important in facilitating engagement with these experiences and shaping how they are interpreted and integrated (18). Subjective experience may therefore be central to understanding how KAP operates.

Emerging research in psychedelic- and ketamine-assisted treatments for substance use disorders suggests that participants often report changes in self-concept, enhanced personal meaning, increased motivation, and altered relationships with substances (17, 19–23). Qualitative studies highlight the importance of subjective experience in KAP, with participants reporting dissociative, mystical-type (e.g. sense of unity, transcendence, ineffability, and connection to others or the broader environment), and meaningful experiences alongside perceived changes in their relationship with alcohol and opioids (17, 18, 24). Although ketamine differs from classic psychedelics in its receptor profile, there are important overlaps in downstream processes related to neuroplasticity and reward learning. Converging evidence suggests that subjective and relational factors may play an important role in shaping therapeutic outcomes alongside these neurobiological processes (11, 12, 25). While qualitative studies of KAP for alcohol use disorder and treatment-resistant depression have begun to explore the role of these processes – including subjective and contextual factors (e.g. therapeutic setting, preparation, therapist support) - in shaping treatment experience and outcomes (17, 26), such investigations remain limited and have not, to our knowledge, examined KAP for methamphetamine use disorder (MAUD).

Methamphetamine is the most widely used synthetic stimulant globally (27), and MAUD represents a significant clinical challenge. Frequent methamphetamine use is associated with substantial psychiatric, cognitive, and physical morbidity, including increased risk of psychosis, cardiovascular disease, sleep disturbance, and neurocognitive impairment (28, 29). Pharmacological treatments are limited (30), and psychosocial interventions, while first-line, show modest and time-limited effects (31). KAP may enhance engagement with psychotherapy by transiently modulating affective and cognitive processes relevant to early recovery (32).

In early-phase trials of interventions involving altered states of consciousness, feasibility and acceptability cannot be fully captured by quantitative outcomes alone. While retention, adverse events, and substance use are important indicators, they may not capture the depth and richness of how participants experience and understand treatment, its perceived credibility or tolerability, or its role in facilitating change. Qualitative inquiry can provide insight into participant meaning-making, perceived mechanisms, and factors influencing engagement, thereby informing intervention refinement and trial design (33). Prior qualitative work embedded within ketamine trials in other contexts demonstrates the value of this approach in elucidating processes not captured by quantitative measures (17, 26). Such methods can clarify how participants understand the interplay between ketamine-induced altered states, psychotherapy, and treatment context in shaping change.

The present study aimed to explore how participants experienced, interpreted, and evaluated KAP for MAUD.

## 2 Methods

### 2.1 Participants and setting

Participants were drawn from a recently completed open-label safety and feasibility pilot trial of KAP for MAUD [the KAPPA trial (32),]. KAPPA is a four-week intervention comprising three subanaesthetic ketamine doses (0.5–0.9 mg/kg, delivered subcutaneously and titrated according to tolerability) administered at weekly intervals, alongside four sessions of cognitive behavioural therapy (CBT; one at treatment initiation and three delivered within 24–48 hours following each ketamine session). The intervention comprised structured preparation, ketamine dosing sessions, and post-dose CBT sessions incorporating integration.

Ketamine dosing sessions were conducted in a dedicated clinical room within the outpatient Stimulant Treatment Program at St Vincent’s Hospital Sydney, providing a private and medically supervised environment. During each session, participants were attended by a registered nurse, with medical oversight available from a study doctor, and were monitored throughout the acute effects period (approximately 2 hours post-administration). Participants were seated or reclining in a comfortable clinical environment, with minimal external stimulation, and were encouraged to focus on their internal experience during dosing.

In line with the KAPPA protocol (32), psychological preparation formed a structured component of the intervention. Preparation was introduced during the initial CBT session and revisited immediately prior to each dosing session, including discussion of expectations, intention-setting, and psychoeducation about the anticipated effects of ketamine. Grounding and relaxation strategies (e.g. breathing exercises) were facilitated by study staff prior to administration, and participants were encouraged to draw on these during dosing if distress or anxiety arose.

CBT sessions delivered following dosing incorporated an integration component, in which participants reflected on their ketamine experiences and linked these to cognitive–behavioural therapeutic content. These preparatory and integrative elements were intended to support emotional processing, enhance engagement with therapy, and provide continuity between pharmacological and psychotherapeutic components of the intervention.

Inclusion criteria for the trial were consenting adults aged 18 years and older, meeting Diagnostic and Statistical Manual of Mental Disorders (DSM)-5-TR criteria for current stimulant use disorder—amphetamine-type substance, and a positive urine drug screen for methamphetamine at baseline. Individuals meeting DSM-5-TR criteria for current or past use disorder for ketamine or ketamine analogues, current other substance use disorder (moderate or severe; except tobacco, caffeine or cannabis), psychotic or bipolar disorder were excluded, as were those with any other medical or psychiatric condition which, in the opinion of the principal investigator, would make participation hazardous (see (32) for full criteria). These exclusions were applied to minimise the risk of adverse psychological or physiological effects associated with ketamine administration.

Participants were included in the qualitative analysis if they had completed at least two CBT sessions and received at least one ketamine dose. This criterion ensured a minimum level of exposure to both components of the intervention, which are understood to act synergistically in KAP, and was considered sufficient for participants to meaningfully reflect on the core therapeutic processes. Participation in qualitative interviews was voluntary, and declining participation did not affect involvement in the trial. Participants were recruited consecutively from those completing core intervention components and consenting to interview. No participants required an interpreter.

The trial and embedded qualitative study were approved by the St Vincent’s Hospital Human Research Ethics Committee (reference 2023/ETH00530). All participants provided written informed consent for trial participation and separately consented to qualitative interviews. Participants received an AU$40 voucher as reimbursement for interview participation.

### 2.2 Design

This qualitative study employed semi-structured interviews to explore participants’ experiences of KAP within the pilot trial. Interviews were conducted during weeks 5–8 (at least one-week post-intervention) by two members of the research team (KF, SVDH). Interviewers were not involved in delivering ketamine or CBT sessions.

Interviews were conducted in the ketamine dosing room used during the intervention, partly to facilitate context-dependent recall of treatment experiences. Interviews were audio-recorded and transcribed verbatim by an independent transcriber. Identifying information was removed prior to analysis.

The interview schedule explored four domains aligned with the study aims of understanding participants’ experiences, interpretations, and evaluations of KAP: (1) treatment entry and expectations, capturing motivations for seeking treatment and contextualising participants’ engagement; (2) overall experiences of participating in the trial; (3) experiences of the intervention, including ketamine dosing sessions and CBT; and (4) reflections on perceived impacts and future directions, which elicited participants’ evaluations of the intervention and its role in facilitating change (see **Appendix**). Participants were encouraged to elaborate freely and to raise additional experiences they considered important.

### 2.3 Analysis

Data were analysed using Reflexive Thematic Analysis (RTA) as described by Braun and Clarke (34, 35). RTA involves identifying and interpreting patterns of shared meaning (themes) through active, reflexive engagement with participants’ accounts. We adopted a primarily realist/essentialist epistemological orientation (34, 35), working on the assumption that participants’ language provides meaningful access to their experiences, while recognising that themes are generated through researchers’ interpretive engagement with the data. Prior literature on KAP informed the broader analytic context but was not used as a pre-specified coding framework; instead, it functioned as a set of sensitising concepts during interpretation.

Analysis was led by the first author (KF) and followed Braun and Clarke’s six-phase approach in a recursive and flexible manner. Transcripts were read and re-read to support data familiarisation, and initial codes were generated through line-by-line coding by KF. Codes were then organised into candidate themes, which were discussed with the wider research team (KF, NE, KJS, JF, BC) to facilitate reflexive analytic dialogue. These discussions focused on exploring alternative interpretations, interrogating assumptions, and refining the conceptual coherence of themes, rather than seeking consensus or inter-rater reliability. Themes were subsequently reviewed and refined in relation to the coded data and the dataset as a whole, before being defined and named to capture their central organising concepts. The final themes were then developed into a coherent and analytically meaningful account of participants’ experiences.

Reflexivity was maintained throughout, particularly through ongoing team discussions, with explicit consideration of how researchers’ multidisciplinary backgrounds (including clinical psychology, addiction medicine, nursing and clinical research in substance use, and public health) and perspectives on KAP and substance use disorder treatment may have shaped interpretation. Analysis was conducted in NVivo (Version 15; Lumivero).

Participants are identified using pseudonyms to protect confidentiality and were informed during recruitment that any published data or quotes would not reveal their identity.

## 3 Results

A total of 14 participants (11 men, 3 women) out of the 17 participants who had taken part in the pilot trial completed qualitative interviews. Those not interviewed either did not complete core intervention components or were unavailable for interview, and no clear differences in baseline characteristics were observed between those who were and were not interviewed. The majority of those interviewed (n = 11, 78.6%) completed the full KAPPA intervention. The median age of participants was 44 years (IQR 38-50.3); half (n = 7, 50%) had completed a university degree; 8 (57.1%) reported relying on social benefits for income. At the time of enrolment in the pilot trial, self-reported methamphetamine use history was collected: median age of first use was 30 years (IQR 21.8-33.5); median duration of regular methamphetamine use was 10 years (IQR = 6.0-15.8); and current use (median days) over the past 28 days (assessed at baseline) was 28 (IQR 22.8-28). When asked ‘What do you think will happen regarding your use of methamphetamine in the next 8 weeks’: 8 participants (57.1%) indicated ‘reduce my use of methamphetamine, but not stop altogether’, 2 (14.3%) indicated ‘stop using methamphetamine altogether’, 2 (14.3%) indicated ‘don’t know’ and the remaining participant (7.1%) indicated ‘keep the amount about the same.’ Five participants (35.7%) reported using ketamine extra-medically within the past year. None met DSM-5-TR criteria for current or past ketamine use disorder, which was an exclusion criterion for trial participation. Full results will be reported in the forthcoming publication of the pilot trial.

### 3.1 Qualitative results

Interviews ranged from 32 to 93 minutes (median 61 minutes, IQR 58.5-66). Four interrelated themes were identified: (1) treatment entry; (2) structured and supportive context for engagement; (3) altered states and psychological shifts; and (4) translation of psychological shifts into behavioural change. Together, these themes describe KAP as a multi-stage process shaped by prior treatment experience, therapeutic context, subjective effects of ketamine, and ongoing efforts to translate these experiences into behavioural change.

#### 3.1.1 Theme 1: treatment entry

Participants consistently described arriving at KAP after multiple prior attempts to reduce or cease their methamphetamine use, often with limited sustained benefit. However, engagement in this trial was not framed simply as another attempt at treatment, but as a shift toward something perceived as meaningfully different. Within a context of prior treatment experience with limited sustained benefit, alongside a growing recognition that ongoing methamphetamine use was unsustainable, KAP was taken up as a novel approach, grounded in its perceived distinctiveness from prior interventions and the possibility that it might operate differently. Importantly, participants framed KAP not simply as another treatment attempt, but as a qualitatively different approach that might operate through novel mechanisms. Across accounts, participants articulated pragmatic treatment goals in relation to methamphetamine use (i.e., reducing vs. ceasing) alongside a cautious openness to engaging with a novel intervention.

##### 3.1.1.1 Nothing else worked

Many participants described extensive prior engagement with different interventions including psychological therapies, psychiatric support and 12-step programs. Despite repeated efforts, most had not achieved sustained change. Paul summarised this history of repeated attempts:

I’ve tried so much different shit … psychologists … psychiatrists. 12-Step programs … Narcotics Anonymous … I’ve done all of them. Reading the basic texts. Reading the big book. And nothing really worked. And so, this was like, you know, “What the fuck have I got to lose?”

These accounts conveyed a sense of treatment frustration given that lack of sustained change. For Luke, KAP represented a novel or “cutting edge” approach, and this perceived distinctiveness contributed to its appeal. Similarly, Casey noted:

“I’d heard about drugs like ketamine, I guess, and that fact that that could have a positive impact on substance use and also depression … and just sort of thought that it might be a good chance to do something … about my [methamphetamine] use…”

Engagement in KAP was therefore situated within a broader trajectory of persistent but frustrated attempts to change but was distinguished by its perceived difference from prior treatment approaches. Trial participation reflected a recalibrated willingness to attempt something meaningfully different.

##### 3.1.1.2 Methamphetamine use as unsustainable

Alongside prior treatment experience with limited sustained benefit, participants described an increasing awareness that their methamphetamine use was no longer consistent with their goals, values, or desired way of living. Ongoing use was often experienced as increasingly difficult to manage and as interfering with personal, occupational, and relational priorities. While some aimed to cease use entirely, others framed their goals more pragmatically in terms of reduction. Morgan reflected:

“It was funny: I didn’t wanna say that I wanted to stop initially because I was afraid I might not get there … I don’t think I put an amount on it but I wanted to not be controlled by it. I’d like to be able to live my life as usual without having to worry about will I have enough meth … So, I didn’t really have like fixed, like concrete goals but I guess I knew that I wanted to do less, at least, or not have to worry about it.”

Andrew described an initial intention prior to treatment to decrease use, to recognising that it “*just has to be full-stop*” as occasional use on weekends was not working for him.

These accounts suggest readiness emerged from growing awareness of personal and social costs, alongside a desire to regain agency. Participants indicated that methamphetamine use was increasingly experienced as constraining rather than enabling, making change feel necessary rather than optional. This growing recognition of the limits of ongoing methamphetamine use appeared to increase willingness to engage with unconventional interventions such as KAP, particularly those perceived as offering a different pathway to change.

##### 3.1.1.3 Trying something different

Participants commonly emphasised entering the trial with an open mind, expressing willingness to engage despite uncertainty about what ketamine would feel like within this context or whether it would lead to change. The dominant emotional tone was curious and cautiously hopeful about a novel approach, but largely uncertain.

For some, the research context itself reduced barriers to engagement. Morgan noted that participating “for science” made it easier to seek help. Andrew commented that the clinical setting, with clinicians present, further legitimised experimentation and mitigated apprehension:

“I was interested to do it ‘cause I was like … this is the environment to do it in. Like doctors, nurses. Like what’s the worst that can happen?”

In this way, the clinical and research context appeared to legitimise engagement with ketamine, framing it as a therapeutic rather than recreational substance and lowering barriers to participation.

Some participants drew on previous psychedelic and ketamine use: prior experience reduced anxiety for some; for others, unfamiliarity increased uncertainty. In general, openness was not predicated on familiarity or enthusiasm for psychedelics, but rather on a broader readiness to engage with an intervention that felt meaningfully different from previous attempts, along with hopefulness for psychological benefit and/or reduced methamphetamine use. Simon commented:

“I was hoping to obviously get, you know, put in the right mindset or like in a different mindset because of it … But I knew that I would probably have to do work afterwards to, you know, achieve my goals…”

Taken together, participants described entering KAP following multiple prior attempts to reduce or cease methamphetamine use, but viewed this intervention as meaningfully different from previous treatment approaches. Participants articulated varied goals, ranging from reduction to ceasing use, and generally approached treatment with cautious openness rather than firm expectations. The structured clinical setting and research context appeared to enhance acceptability and support engagement with this novel intervention.

#### 3.1.2 Theme 2: structured and supportive context for engagement

This theme focuses on the external conditions that shaped engagement with KAP, including relational, procedural, and environmental factors. While participants came into treatment with varied goals and differing expectations, their accounts indicated that the structured and relational context in which KAP was delivered shaped how they engaged with the intervention. Although participants often referred to features of the clinical trial (e.g., monitoring procedures, staff contact, structured scheduling), these were not experienced as separate from the intervention itself, but as integral to how KAP was delivered and made tolerable, particularly given the intensity of ketamine-induced experiences. Across interviews, the hospital setting, preparation procedures, and ongoing contact with trial staff were described not merely as administratively necessary, but as fostering safety, legitimacy, and accountability. These features were commonly experienced as supportive rather than intrusive or overly medicalised, enabling participants to engage with a novel and potentially confronting intervention such as KAP. Engagement was not attributed solely to individual motivation, but also to the structured and relational conditions within which KAP was delivered. While many of these features are not unique to KAP, participants described them as important in enabling engagement with ketamine-induced altered states.

##### 3.1.2.1 Feeling valued and supported

Participants consistently described the broader trial environment as welcoming and respectful. Tom contrasted this experience with prior help-seeking experiences, where he had felt judged:

“Everyone here was very, very accepting and easy to get along with … There was never a feeling of judgement … which happens a lot when you’re on meth.”

Accounts consistently emphasised warmth, professionalism, and responsiveness, suggesting that the relational tone of the setting facilitated openness and participation. This relational tone appeared to reduce defensiveness and shame, supporting participants to engage more fully with the intervention. Paul commented:

“[The research nurse] was really easy to kind of, you know, work with and also really flexible and accommodating, which kind of made me … want to keep my commitments to come here, even though it was a really busy time for me at work.”

Monitoring procedures and observation during ketamine administration were not framed by participants as surveillance, but as reassurance. This appeared particularly important in the context of ketamine administration, where monitoring and staff presence were interpreted as supporting safe engagement with an unfamiliar and potentially intense experience. Jamie described the presence of the research nurse in the room during the ketamine session as “*comforting … because after I had that really intense experience at the start … she was … just reassuring me that it’s okay*.”

The presence of medical staff signalled protection and legitimacy rather than control. As Morgan noted:

“I was quite familiar with [the research nurse] … I’d met her several times … I don’t necessarily know that [ketamine] is a safe drug to do … in whatever dose we had it … in a way it felt like … giving up control over my body and my … mental experience … I wouldn’t necessarily have been comfortable with doing it unsupervised.”

In this context, respect, support and relational validation appeared central to intervention engagement.

##### 3.1.2.2 Relational safety

Participants spoke specifically about the quality of their relationships with the therapists delivering the CBT sessions, with rapport and non-judgement repeatedly described as essential. As Daniel stated, “*You’ve gotta have the rapport, the respect … between you and the counsellor or they’re ineffective*.”

This was reflected in participants’ descriptions of their own therapeutic relationships. Jordan found his therapist “*insanely easy to talk to*”, while Paul described his therapist as “*very non-judgemental … made me feel like I can tell him anything*”.

These accounts foreground the importance of therapeutic alliance in supporting engagement, particularly in the context of methamphetamine use, where prior experiences of stigma and shame were commonly described. Being listened to without judgement appeared to enable more open engagement with the intervention.

Even when participants expressed scepticism about CBT as a modality, they distinguished this from their evaluation of the therapist. Several noted, like Jordan, that although the content sometimes felt familiar or “*basic*”, the therapist’s manner - engaging, pragmatic, and respectful - made sessions feel worthwhile. Jordan commented:

“None of it was new material to me … It was delivered by an excellent counsellor. Pretty professional. Smart. Engaging … It wasn’t a disappointment; it was just … this is CBT.”

This relational safety appeared to function as a foundation for engagement. Participants described being able to speak honestly, disclose difficult material, and remain in conversation without feeling judged. While most clearly articulated in relation to the therapist, similar experiences of respect and non-judgement were also described in interactions with research and clinical staff, suggesting that relational safety operated across the broader treatment environment.

##### 3.1.2.3 Preparation and due care as reassurance

Preparation procedures prior to dosing were largely experienced as responsible and comforting. This preparation appeared to play a key role in enabling participants to approach ketamine sessions with sufficient confidence to engage with potentially disorienting or intense experiences. Tom described the emphasis on medical oversight and safety checks as evidence of due care:

“It felt responsible and comforting to know that there was … a lot of due care … you covered all the bases.”

Grounding strategies and preparatory discussions were generally described as helpful, even when not always fully utilised. Casey expressed a desire for more detailed preparation:

“I thought that there probably could have been more guidance on what to expect maybe, in terms of what it might feel like … maybe more focus on the grounding techniques before each dose.”

Morgan indicated:

“It felt like precautionary rather than like something I might actually need at the time … once I had the experience, I was like … I didn’t really need to worry about any of the grounding exercises or anything, because I felt very comfortable with where it went, I guess. Whereas the second time … I was just absolutely flying off the wall sort of thing for most of it. So … even though I don’t remember it that well, I do remember the grounding exercises being helpful.”

Where preparation was perceived as insufficient, this appeared to relate to uncertainty about how to navigate the subjective effects of ketamine, rather than concerns about safety per se. However, these reflections did not undermine the broader sense of safety reported by participants. Overall, preparation appeared to signal that the intervention was structured, deliberate, and therapeutically contained rather than experimental or unpredictable.

##### 3.1.2.4 Structured accountability

Although trial participation required frequent visits, questionnaires, and scheduled appointments, these were rarely framed as burdensome. Instead, several participants described the structured nature of clinic attendance as beneficial. Daniel noted:

“I think the discipline … is good as well because … increased meth use … leads to lackadaisical behaviour … I think it’s good from that discipline perspective to have an appointment … to get back in tune with the way the world works.”

Physically attending sessions required preparation, punctuality, and commitment - behaviours that participants associated with re-establishing routine. This structure appeared to support ongoing engagement and, for some participants, may have facilitated efforts to translate psychological shifts associated with KAP into behavioural change through regular contact and accountability.

Reflections on online and group formats further underscored the perceived value of structure. Many participants emphasised that face-to-face engagement fostered accountability and commitment in ways that fully remote formats might not, and for several, the act of showing up in person itself was part of the work of change, particularly in maintaining engagement during the period following ketamine sessions. Tom commented:

“Personally, in person is better … having to go and meet someone and be there forces that level of commitment … whereas I think online I could easily fall into ticking a box, rushing through it … The commitment to me personally means that I am committed.”

Taken together, participants described the structure of the trial not as burdensome or peripheral, but as central to their ability to engage with the intervention. The predictability of appointments, preparation and medical oversight during ketamine dosing, and ongoing contact with clinicians were experienced as reassuring and legitimising, reinforcing a sense that this novel treatment was deliberate and carefully delivered. For many, the requirement to attend in person and participate consistently introduced routine and accountability that contrasted with patterns of disrupted routine associated with methamphetamine use. Even where elements of the therapeutic content felt familiar, the relational tone and structured delivery of the intervention was described as sustaining participation. These findings suggest that, in the context of KAP, relational and procedural elements are not peripheral but may play an important role in supporting participants to engage with altered states and, for some, to translate these experiences into ongoing therapeutic work. While many of these features - such as therapeutic alliance, structured engagement, and non-judgemental care - are characteristic of high-quality substance use treatment more broadly, participants described them as particularly important in enabling engagement with the altering and sometimes challenging subjective effects of ketamine. In this context, relational and procedural elements appeared to function not only as general supports to treatment engagement, but as conditions that made participation in the intervention feel safe, legitimate, and tolerable.

#### 3.1.3 Theme 3: altered states and psychological shifts

Participants described considerable variability in the intensity and quality of ketamine-induced experiences, both between individuals and across dosing sessions. However, across this variation, many accounts converged around a shared sense of ‘psychological space’. We use the term ‘psychological space’ to describe a subjective sense of distance from habitual emotional and cognitive responses, enabling reflection and reduced automatic reactivity. Participants described ketamine sessions as producing marked alterations in perception, bodily awareness, and emotional experience, and often framed their impact not in terms of cessation of methamphetamine use per se, but as internal shifts in how they experienced themselves, their thoughts, and their emotions. This shift was often described as introducing a pause between trigger and response, which participants linked to reduced impulsivity and craving.

##### 3.1.3.1 Altered perception and intensity

Experiences during dosing sessions were described as vivid, unusual, and difficult to articulate. Participants used imagery of dissociation, altered perspective, and sensory distortion to convey the intensity of the state. Jordan described his first session as a “*multi-sensory, hallucinogenic wonderland*”, while Morgan reflected that her brain was “*doing something it doesn’t normally do*”.

Several participants described sensations of separation between mind and body, or a shift in perspective. Casey described:

“It kind of felt like … a separation between brain and me … like physically not contained.”

For some, these experiences were described as peaceful or beautiful, while for others they were intense or at times frightening. Jordan described his experience as “*profoundly spiritual … otherworldly … beautiful*”, while Jamie commented “*Look, to be honest, it was hell … it was really difficult.”*

Importantly, even challenging moments were often framed as part of the process rather than inherently harmful. As Luke described:

“…you start to think you’re opening up parts of your brain that aren’t meant to be … [they] were shut for a reason. And … when you accept the fuckin’ good stuff coming through, you’ve gotta accept the bad.”

Participants, like Luke, described consciously leaning into the experience “*ride it out … let go*”, or reminding themselves that the experience was purposeful. Jordan described this process:

“First impression I’d be like, ‘Whoa! Intense!’ which is when I made the very conscious decision to remind myself that this is for me. Yeah. This is not a punishment: this is a gift.”

Across accounts, ketamine was also positioned as qualitatively distinct from prior drug use. Even participants with previous psychedelic exposure emphasised that it felt unlike other substances in terms of its effects. This perceived distinctiveness appeared to shape how participants understood the experience, often framing it as something more deliberate or meaningful than recreational intoxication. Michael described:

“…it clearly works an entirely different level to whatever anything psychedelic is doing … [there were] no real changes in thinking that I’d noticed during the [ketamine] experience, which again was completely different to the psilocybin. In the days afterwards … I found myself having a couple of ideas about things that I’d thought about a lot but in drug use … that were a bit out of left field, that I had never really encountered before. And it wasn’t really until after the fact that I thought, maybe this is part of, like bits of my brain reorganising some like bits in new places or connecting bits that weren’t previously connected.”

##### 3.1.3.2 Psychological space: shifts in emotional and cognitive reactivity

While the experiential intensity of dosing sessions was frequently noted, participants more consistently emphasised changes in emotional tone and reactivity in the days following ketamine administration. In several accounts, the most salient effects were not dramatic insights but a softening of emotional volatility and a sense of increased psychological space – experienced as feeling calmer, more present, and less immediately reactive to internal and external stressors.

For example, several participants described feeling calmer or more regulated. Alex reflected that after dosing she experienced a “*significant feeling of clarity*,” describing her chest as feeling “*more open … for about two, three days*”. Simon described the experience as providing “*an overwhelming sense of peace … just like being able to take a breath of fresh air*”. Paul framed this shift as his “*brain being quieter*”, while Alex described feeling “*more present*” in everyday situations.

For some, this shift was described as a reduction in emotional intensity in response to stressors. Situations that would previously have provoked distress or spiralled into prolonged rumination felt more manageable. Morgan commented:

“I think it’s helped me to not worry about the issues that have caused me to use methamphetamine … my family issues … things that would have destroyed my day. Now … they don’t destroy my week sort of thing … I’m less bothered by them.”

Similarly, Jamie described feeling less impulsive and less driven to react automatically:

“I’ve had like a month off [methamphetamine] before and I haven’t felt as clear-minded … the ketamine kind of … in my brain like … just slowly putting it back together, it felt like … and it kind of made me feel like less impulsive … I’m usually, yeah, very, very impulsive. And … very quick to say yes and use … But I haven’t felt that part of me since … It’s nice, yeah.”

Several participants also described changes in their relationship to methamphetamine use in the days following dosing. For some, this was experienced as a reduction in the immediacy or urgency of craving. Simon reflected:

“Well, it definitely decreased my like thoughts that, to want to use … and I, I felt as if that like I could like stop…”

Jamie described having “*zero cravings … it’s been fuckin’ wild*”, noting that even when confronted with familiar triggers, the pull to act felt less compelling, at least in the days following dosing.

Rather than describing a complete absence of desire, participants more commonly depicted a shift in intensity - a pause between emotional trigger and behavioural response of drug use. Situations that had previously led quickly to use were experienced as more manageable, or at least less urgent.

However, these effects were not universally experienced nor consistently sustained. Several participants expressed uncertainty about whether these shifts could be attributed solely to ketamine, therapy, personal motivation, or the broader structure of the trial. Others described the changes as transient, fluctuating over days rather than signalling a permanent transformation.

For some, this psychological space was experienced as a reprieve from entrenched patterns. Morgan described feeling “*less concerned*” and “*more detached*” from issues that had previously triggered her methamphetamine use, noting that while the problems remained, she was “*less bothered by them*” day-to-day. In this way, ketamine was described not as removing stressors, but as altering the immediacy and intensity with which they were experienced.

Taken together, these accounts suggest that many participants experienced a temporary attenuation of habitual emotional and cognitive reactivity. This attenuation was experienced as creating a psychological space - characterised by calm, clarity, and reduced urgency – that introduced a degree of separation between trigger and response. Participants frequently contrasted this with their typical patterns of reactivity, while also acknowledging its variability over time and its impermanence.

#### 3.1.4 Theme 4: translation of psychological shifts into behavioural change

While many participants described ketamine as creating psychological space (Theme 3), accounts of behavioural change were more variable and often framed as emerging from the combination of ketamine and CBT rather than from the ketamine in isolation. Participants described reductions in methamphetamine use, shifts in motivation, and increased engagement with therapeutic material, but frequently emphasised that these outcomes were contingent and multi-determined rather than automatic.

##### 3.1.4.1 Observable shifts in use

Several participants described measurable reductions in methamphetamine use during or following the trial. For some, this was experienced as a gradual decrease rather than abrupt cessation. Daniel commented:

“There has been … a reduction in my consumption … I’ve got to a point and realised I’ve had virtually nothing … it is decreasing.”

Jamie described more dramatic shifts:

“Well, I don’t smoke meth anymore … I’ve just gone cold turkey on it.”

However, even among those reporting substantial reductions, attribution was often cautious. Daniel reflected:

“What is that attributable to? I really don’t know. I can’t say 100 per cent it was the ketamine.”

For others, reductions were partial or temporary. Tom commented:

“So, there was definitely a reduction but I, yeah … I can’t deny that I’ve pretty much reverted back to where I was. So, it’s helped reduce but it’s definitely not helped stop.”

Chris felt that the CBT sessions had no impact on his methamphetamine use, but “*it was definitely a foot in the door compared to anything else I’ve experienced in the past*”. When asked what he attributed this to, he commented “*because of the ketamine … or maybe [the therapist] … I don’t know*.”

Across accounts, behavioural change was described less as a uniform outcome and more as a shifting trajectory influenced by multiple factors. However, not all participants experienced meaningful reductions in methamphetamine use. Some described minimal change, while others reported that early reductions were not sustained over time, highlighting the variability of outcomes within the sample.

##### 3.1.4.2 Receptivity and consolidation: the value of the combined approach

A common, though not universal, pattern was the framing of ketamine and therapy as interdependent components of a broader therapeutic process. Participants frequently described ketamine as creating a state of receptivity that altered how they engaged with CBT sessions over the following days. Simon described feeling:

“Very grounded and present … like an after-glow sort of thing. No brain fog … no, like, anxiousness where it’s like, you know, I’ll be thinking about it too hard. It was just like, yeah, like present, grounded and, yeah, just on it.”

Jordan felt similarly:

“I just felt receptive and responsive and efficient without haste … very functional and eager.”

Luke reflected that, following ketamine, “*whatever information was thrown to me, I was absorbing it all*”, and for Paul, this translated into increased motivation to engage with materials and apply strategies in practical ways:

“Instead of going, ‘Oh, these are the strategies,’… it became practical. Because now I’m applying it to my own life.”

Many participants described ketamine and psychotherapy as interdependent components of change. Paul articulated:

“The ketamine only gives your brain an opportunity to absorb the treatment … the treatment is the CBT.”

Luke described the interaction more bluntly:

“The K will get you there … but it’s … the psychotherapy that really fuckin’ nails it down.”

In this framing, ketamine was understood as facilitating engagement, while therapy provided structure, tools, and accountability necessary for consolidating change. The intervention was therefore frequently described as synergistic rather than additive: ketamine was often described as creating a period of receptivity, and therapy as shaping how that period was used. However, it is not possible to determine the extent to which these accounts reflect participants’ own interpretations versus expectations shaped by the rationale of the intervention as explained during the trial.

##### 3.1.4.3 Variability, limits and attribution

Despite accounts of methamphetamine reduction and increased engagement with CBT, participants were generally cautious about permanence and causality. Some explicitly questioned whether changes were attributable to ketamine, therapy, personal readiness, contextual timing, or expectancy effects. Paul commented:

“Maybe the ketamine helped … but maybe I wanted it to work so much…”

Notably, participants differed in how they explained these changes: some attributed shifts primarily to ketamine, others to therapy, and others to their own readiness or contextual timing. For example, Alex reflected that improvements occurred for her within the broader context of intentional effort:

“It’s hard to know [if the intervention worked] because, well, I have reduced [methamphetamine] and did reduce it gradually throughout the trial. I’d sort of had the plan to use the trial as a time to hopefully stop for a period.”

Several noted that while reductions occurred during the structured period of the trial, sustaining those gains required ongoing support. Tom noted:

“More consistent counselling afterwards would definitely have that fresh in my mind.”

This variability underscores that behavioural change was not experienced as an automatic consequence of altered states. Rather, participants described an interaction between psychological shifts, therapeutic engagement, personal motivation for change, and the trial structure.

Overall, participants described KAP as creating an opportunity for change rather than guaranteeing it. For some, this opportunity translated into substantial reductions in methamphetamine use; for others, it resulted in partial, temporary, or minimal change. Outcomes were described as emerging through an interaction between psychological shifts, therapeutic engagement, personal motivation, and contextual factors, rather than as a direct or uniform consequence of ketamine administration. While many participants viewed the combined approach as more meaningful than either component alone, this perception was not universal and remained subject to uncertainty and variability.

## 4 Discussion

This study provides the first qualitative account of KAP for MAUD. Findings suggest that KAP is experienced as a multi-stage, context-dependent process in which pharmacological, psychological, and contextual factors interact over time. The four themes generated in this study suggest that ketamine was experienced not as a stand-alone treatment, but as one component of a broader therapeutic process involving subjective experience, psychotherapy, and treatment context. Participants described transient shifts in emotional and cognitive reactivity occurring within a structured therapeutic environment, with variable translation of these shifts into behavioural change. However, participants did not always describe these components as clearly integrated. While some accounts emphasised interactions between ketamine and psychotherapy, others foregrounded the ketamine experience itself, and many expressed uncertainty regarding the relative contribution of different elements of the intervention.

**Figure 1:**
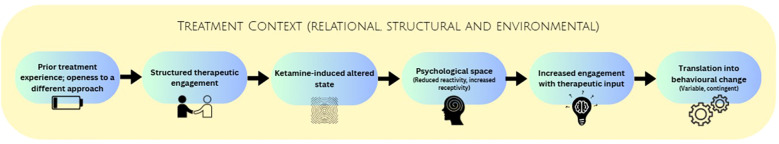
Provisional theory of change for ketamine-assisted therapy in methamphetamine use disorder. KAP is conceptualised as a multi-stage, context-dependent process. The shaded box represents the overarching relational and structural context within which all stages occur. Participants enter treatment with varying levels of readiness and prior treatment experience and engage within a structured therapeutic environment. Ketamine administration induces an altered state, followed by the emergence of a subjective sense of ‘psychological space’, characterised by reduced emotional and cognitive reactivity and increased receptivity to internal and external experience. This is associated with enhanced engagement with therapeutic input, which may support behavioural change. Outcomes are variable and contingent on individual, therapeutic, and contextual factors. Features of the clinical and relational context are conceptualised as active components influencing multiple stages of the process. This model should be understood as heuristic rather than strictly sequential, reflecting common patterns across participant accounts rather than a uniform or deterministic pathway. Individual trajectories varied considerably, and not all participants described progression through each stage in a linear manner.

We propose a provisional, context-specific theory of change for KAP in MAUD (**Figure 1**), synthesising the four themes into a process model of how participants described the intervention as unfolding over time. These themes map onto different stages of the intervention: treatment entry; engagement within a structured and supportive context; ketamine-induced altered states and the emergence of psychological space; and the translation of these shifts into behavioural change.

This model comprises six interrelated stages: entry into treatment, often characterised by prior treatment exposure and openness to novel approaches; engagement within a structured and relationally safe environment; induction of a ketamine-induced altered state; emergence of a subjective sense of psychological space, characterised by reduced emotional and cognitive reactivity; a period of increased receptivity to therapeutic input; and the variable translation of these shifts into behavioural change. While several of these processes are well described across substance use treatment more broadly, this model highlights the potential role of pharmacologically induced altered states in creating a temporally bounded window of psychological flexibility and receptivity within KAP. **Figure 1** provides a visual summary of this process, illustrating how altered subjective experience, therapeutic engagement, and context interact across stages rather than operating as discrete components.

A key finding was that ketamine was commonly experienced as producing a temporary shift in internal state rather than direct or durable behavioural change. This is consistent with previous qualitative research describing ketamine treatment as a process involving transient alterations in perception, emotion, and self-experience that require ongoing integration and therapeutic support (18). Participants described reduced reactivity, increased emotional regulation, and a sense of cognitive or affective distance from previously dominant patterns. This corresponds to what we refer to here as ‘psychological space’ - a temporary reduction in habitual emotional and cognitive reactivity that enabled reflection and reduced automatic responding. These effects align with emerging literature suggesting that ketamine and related interventions may transiently enhance psychological flexibility, attenuate negative affective bias, and facilitate a more reflective or “observer” stance (36–39). Within substance use disorders, such attenuation of cue-driven responding may create a window in which entrenched patterns are less dominant and engagement with corrective learning becomes more possible (10).

The construct of psychological space also bears conceptual similarity to processes described in Acceptance and Commitment Therapy (ACT), particularly psychological flexibility and cognitive defusion and acceptance-based responding, as well as decentering processes described in mindfulness-based approaches (38). Although participants did not use this terminology, their accounts of reduced attachment to distressing thoughts, feeling more present and less reactive to stressors, and experiencing a greater pause between trigger and response resemble these established psychological processes. We therefore conceptualise psychological space as a phenomenological description derived from participants’ accounts, while recognising its potential overlap with broader transdiagnostic mechanisms that may support behaviour change across a range of mental health and substance use conditions.

Notably, participants did not consistently link these internal shifts to psychotherapy, often describing them primarily in relation to the ketamine experience. However, these shifts were not typically experienced as inherently transformative. Rather, they were understood as creating an opportunity for change that required active engagement and consolidation. Many participants described ketamine and psychotherapy as interdependent components: ketamine was described as facilitating receptivity - enhancing attention, reducing defensiveness, and increasing openness - while therapy provided the structure, tools, and accountability necessary to translate this receptivity into action. This interpretation is consistent with prior literature suggesting that psychotherapy may prolong and enhance ketamine’s effects, with combined approaches associated with improved outcomes compared to pharmacological treatment alone (11, 14, 40). However, sustaining gains appeared to require ongoing support beyond the trial period, reinforcing the view of KAP as creating a window of opportunity rather than a deterministic treatment effect.

Behavioural outcomes were variable and often uncertain. While some participants reported substantial reductions or cessation of methamphetamine use, others described partial or transient change. Attribution was frequently ambiguous, with participants questioning the relative contribution of ketamine, therapy, personal readiness, and contextual factors. These accounts underscore that KAP was not experienced as a uniformly effective intervention, but as a contingent process shaped by multiple interacting influences. Consistent with emerging evidence, the durability of ketamine’s effects appears to depend on consolidation and ongoing support, and acute psychological shifts may not reliably translate into sustained behavioural change without continued therapeutic engagement (11, 14). As a qualitative study embedded within a small open-label pilot trial, these findings cannot determine efficacy or isolate the contribution of individual components. Participants’ accounts may also have been influenced by expectancy, motivation, and the structure of trial participation. Further randomised controlled trials are required to clarify efficacy and mechanisms.

The findings also highlight the central role of context in shaping both experience and outcome. Participants described the clinical environment, therapeutic relationships, and procedural structure as integral to their ability to engage with the intervention. These features were experienced as fostering safety, legitimacy, and accountability, enabling engagement with a novel and sometimes challenging intervention. This aligns with broader literature on “set and setting” in psychedelic research, as well as perspectives emphasising that drug effects emerge through interactions between pharmacology, expectation, and social context (41–45). The broader institutional context of the clinical trial - including regular contact, monitoring, and opportunities for reflection - may also have contributed to engagement. However, it remains difficult to disentangle the influence of research-related structure from clinical components of care.

Acceptability appeared to emerge less from enthusiasm for ketamine itself and more from the interaction between altered subjective experience and a structured, supportive environment. Participants consistently emphasised feeling respected, listened to, and not judged, with these relational features central to their willingness to engage. While ketamine experiences were often described as meaningful, they were also sometimes intense or challenging; however, such experiences were generally framed as tolerable within a supportive therapeutic context. Consistent with prior qualitative work, KAP may evoke vulnerability and the emergence of distressing material, underscoring the importance of preparation, support during dosing, and post-session integration (26). Many of the factors identified - such as therapeutic alliance and structured engagement - are well established in substance use treatment more broadly. The extent to which these processes are uniquely amplified by ketamine remains unclear, but findings suggest that altered states may interact with these established mechanisms in ways that warrant further investigation.

Participants’ accounts of structured engagement as supportive align with broader evidence linking retention and engagement to improved outcomes (46). In stimulant use disorder, structured behavioural approaches such as contingency management demonstrate robust effects on retention and use outcomes (47, 48). Elements often associated with research participation - such as frequent monitoring and scheduled visits - may therefore contribute to behaviour change by supporting accountability and routine. This raises important considerations for trial design, where intervention effects and trial-related structure may be difficult to disentangle. Participants’ experiences reflect KAP as delivered within a clinical trial rather than routine care, and elements such as structured follow-up and research framing may have supported engagement. These contextual influences highlight the often-under-recognised role of clinical research staff in shaping participant engagement, retention and treatment experience. While not specific to KAP, such relational and organisational factors may play an important role in treatment delivery and should be considered in future research and implementation.

Some participants expressed ambivalence regarding CBT as the primary therapeutic modality. While therapists and relational aspects of care were consistently valued, CBT content was sometimes described as familiar or insufficiently aligned with the intensity of ketamine-induced experiences. Participants often distinguished between the value of the therapeutic relationship and the modality itself. This raises questions regarding optimal psychotherapeutic approaches to accompany altered states. While structured cognitive approaches may support consolidation, more experiential or integrative approaches (e.g. Acceptance and Commitment Therapy, integrative psychotherapy) may better engage affective and meaning-oriented processes (38, 49, 50). At the same time, emerging evidence suggests that structured approaches can be effectively adapted for use with ketamine (40). These findings highlight the need for further research into how different therapeutic models can be optimised to capitalise on ketamine-induced receptivity.

Overall, these findings support a conceptualisation of KAP as a facilitative rather than curative intervention. Ketamine appears to create a temporary alteration in psychological processing that may enhance engagement with therapeutic work but does not in itself produce sustained behavioural change. Instead, outcomes emerge through the interaction of altered states, therapeutic input, individual readiness, and environmental context. This interpretation aligns with qualitative findings in related populations, where ketamine-assisted interventions are associated with shifts in meaning, identity, and motivation that require integration within broader recovery processes (17, 24, 26).

From a clinical and research perspective, these findings suggest that future work should focus on supporting the translation of transient psychological shifts into sustained behavioural change. This may include extending post-dosing support, refining integration-focused interventions, and tailoring therapeutic approaches to align with the experiential properties of ketamine. Greater attention to broader environmental context - including social support and living conditions - may also be important, as participants’ accounts suggested that the translation of psychological shifts into behavioural change was contingent on factors beyond the intervention itself. Longitudinal qualitative and mixed-methods research will be critical in understanding how early changes evolve over time and what factors predict sustained benefit.

In summary, participants experienced KAP as creating a temporary shift in psychological processing - characterised by increased openness, reduced reactivity, and greater reflective capacity - within a structured and supportive context. These shifts were not inherently transformative and did not reliably translate into sustained behavioural change without ongoing engagement and support. KAP is therefore best understood as a context-dependent and relational intervention in which subjective experience, psychotherapy, and environment interact to shape outcomes.

### 4.1 Limitations

Several limitations should be considered when interpreting these findings. First, as a qualitative study designed to explore subjective experience rather than test efficacy, the aim was to generate in-depth, contextually situated understandings of how KAP was experienced within this cohort. Transferability should therefore be considered in relation to contextual similarity rather than assumed. In addition, the structured and supportive context of a clinical trial may itself have enhanced engagement and acceptability, potentially limiting transferability to less resourced treatment settings.

Second, the sample was predominantly male, did not include First Nations participants, and comprised individuals willing to enrol in a clinical trial involving ketamine. This may limit the extent to which findings reflect more diverse or less treatment-engaged populations. Our sample size and gender composition did not permit meaningful exploration of whether experiences of ketamine-induced states, therapeutic engagement, or perceived mechanisms of change differed by gender. Future studies should examine these questions in more diverse samples. As the research question sought to understand participants’ experiences of KAP, the qualitative sample was drawn from individuals who completed core intervention components; however, this may over-represent those who were more engaged with or receptive to the intervention. Perspectives of individuals who did not enrol in the trial or declined participation in interviews were not captured, introducing potential selection bias toward more engaged or treatment-responsive participants.

Third, interviews were conducted relatively soon after completion of the intervention. While this supported recall of dosing sessions and immediate psychological effects, it limits conclusions regarding the durability of behavioural change and longer-term integration. In addition, interviews were conducted in the same room used for ketamine administration to facilitate context-dependent recall of treatment experiences. While this may have enhanced participants’ ability to recall and reflect on their experiences, it may also have influenced accounts through contextual cueing, environmental priming, or demand characteristics. Participants frequently described the room as a safe and supportive space, and it is possible that these associations shaped how experiences were remembered and narrated. Participants frequently described perceived benefits as transient or dependent on ongoing support, and the relatively short duration of the intervention may have limited opportunities to consolidate change. Longitudinal qualitative research would provide a more comprehensive understanding of how early experiential changes are sustained, modified, or lost over time.

Fourth, the multi-component nature of KAP limits the ability to disentangle the relative contribution of ketamine, psychotherapy, and contextual factors. Participants described change as emerging from the interaction of these elements, and the present findings therefore reflect the experience of a combined intervention rather than isolating the effects of ketamine itself. Similarly, expectancy effects and participant motivation may have influenced interpretations of change, particularly within a research context in which participants were aware of the aims and proposed mechanisms of the intervention. Prior extra-medical ketamine exposure may also have influenced participants’ expectations, familiarity with ketamine-induced states, and interpretations of subjective experiences.

Finally, participants’ accounts were generated within a structured clinical trial environment in which therapeutic rationales were explicitly discussed. Participants were briefly introduced to the rationale underpinning KAP, including proposed relationships between ketamine, psychotherapy and behavioural change. Consequently, participants’ interpretations may have been shaped not only by direct experience but also by the explanatory frameworks made available within the intervention. Consistent with our reflexive epistemological position, these accounts can therefore be understood as co-produced through interactions between participants, clinicians, and researchers, rather than as direct reflections of underlying mechanisms. These factors may have influenced both participants’ experiences of the intervention and their interpretations of change.

Taken together, these limitations indicate that the findings provide insight into early subjective experiences and perceived mechanisms of KAP, rather than definitive conclusions regarding long-term efficacy or causal pathways.

## 5 Conclusion

This study provides novel qualitative insight into how KAP is experienced by individuals with MAUD. Participants described KAP as creating a temporary psychological space - characterised by reduced reactivity and increased receptivity - that supported engagement with therapeutic work but did not in itself produce sustained behavioural change. Instead, outcomes were experienced as dependent on ongoing effort, therapeutic support, and broader contextual factors.

These findings support conceptualising KAP as a facilitative, context-dependent intervention rather than a stand-alone pharmacological treatment. Future research should prioritise strategies to translate transient psychological shifts into sustained behavioural change and clarify how best to integrate pharmacological and psychotherapeutic components.

## Data Availability

The datasets presented in this article are not readily available due to ethical and confidentiality restrictions. Requests to access the datasets should be directed to k.fletcher@unsw.edu.au.

## Appendix Interview guide

This is a semi-structured interview and is presented as a guide only; participants and the interviewer will be able to lead the line of inquiry throughout the interview to ensure that participant’s thoughts and opinions are accurately reflected.

### Section 1: motivation to seek treatment

- Can you tell me a little about what led you to come here for treatment?
- What are your treatment goals in relation to your methamphetamine use?
- What made you decide to take part in this trial specifically?

### Section 2: general experiences of being in the trial

- In general, how would you describe your experience of being in this trial?
- Was there anything you enjoyed about being in the trial?/Anything you found particularly valuable or useful?
- Was there anything that didn’t work for you? (e.g. too many questionnaires, medical observations, etc.)
- What were your experiences of being asked to complete regular urine drug screens?
- How would you describe your experiences of coming into the hospital for the visits during the trial?

### Section 3: experience of the intervention itself

#### Medication

- Did you have any expectations about what ketamine would do when you agreed to participate in the trial? (prompt: change in consumption, anything else)?
- What did you think about how we gave you the ketamine? (route of administration, dosage frequency)
- How did you feel being given the ketamine in the clinic and while being observed by study staff?
- Before the first ketamine dose, the study nurse spent some time with you to prepare for the dose. What did you think about this?
- Can you describe your experiences during the ketamine session itself (prompt: liked aspects, disliked aspects)?
- o Was the experience similar each time you were given ketamine?
- o Any more positive or negative experiences you’d like to report?
- In the week following each ketamine session, did you find that the effects had any lasting effects (e.g. after the session, in the days following, etc.)
- Do you think the ketamine made any impact on your methamphetamine use? In what way? (Decrease, increase, no change. Describe any changes.)
- Do you think you experienced any side effects from taking the ketamine?
- Do you think you experienced any benefits from taking the ketamine?
- Is there anything else you’d like to tell us about your experience taking the ketamine?
- If ketamine was available to you as a treatment option, would you want to use it? Why or why not?

#### Therapy

- How would you describe your experience of the therapy sessions? What did you think about how we delivered this? (face-to-face, weekly therapy sessions)?
- Was there anything that you particularly liked about the therapy sessions?
- Was there anything that you didn’t like about the sessions?
- How do you think these sessions could be improved?
- Do you think the sessions made any impact on your methamphetamine use? In what way? (Decrease, increase, no change. Describe any changes.)
- Did you experience any other benefits from the sessions?
- Is there anything else you’d like to tell us about your experience with the sessions?

#### Combination of ketamine and therapy

- What are your thoughts about the approach that we used – combining the ketamine with the therapy sessions?
- o What were your experiences participating in the therapy session 24–48 hours after having the ketamine?
- o Do you think that that ketamine helped or didn’t help your engagement with the therapy session? (probe: if helped, in what ways; if didn’t help, thoughts on this)
- Do you think this approach (ketamine to help stop/reduce/manage use of methamphetamines, combined with therapy) would help you meet your treatment goals? Why or why not?
- Have you been using methamphetamine since you finished participating in this trial? (YES/NO) – if yes, when did you start? What do you attribute this to?

### Section 4: thoughts on future research plans

- How would you feel if the therapy sessions were run online (either for you to do in your own time, on your own; or combined with help from a therapist)?
- What about if therapy were run as part of a group (like SMART recovery), what are your thoughts on that?

#### DEBRIEF

- Is there anything else you would like to report related to your experience taking part in the KAPPA trial?
- Do you have any questions?
